# Association Between Uric Acid to HDL-C Ratio and Liver Transaminase Abnormalities: Insights from a Large-Scale General Population Study

**DOI:** 10.3390/medicina61081417

**Published:** 2025-08-05

**Authors:** Abdulaziz M. Almuqrin, Mousa H. Muqri, Ahmed M. Basudan, Yazeed Alshuweishi

**Affiliations:** Department of Clinical Laboratory Sciences, College of Applied Medical Sciences, King Saud University, Riyadh 12372, Saudi Arabia

**Keywords:** uric acid, HDL-cholesterol, ALT, AST, UHR, Saudi Arabia

## Abstract

*Background and Objectives*: The uric acid to HDL-cholesterol ratio (UHR) has recently emerged as a promising biomarker reflecting systemic inflammation and metabolic disturbances. Elevated liver transaminases are clinical indicators of hepatic injury and underlying metabolic dysfunction. Many Middle Eastern countries face constrained clinical and laboratory resources, where access to comprehensive diagnostic tools may be limited. In such settings, identifying simple and easily accessible markers could offer significant practical value in detecting and monitoring health disorders. This study investigates the potential association between UHR and elevated liver transaminases levels in the Saudi general population. *Materials and Methods*: This retrospective cross-sectional study included 9618 subjects, and the association between the UHR and elevated liver transaminases, alanine transaminase (ALT), and aspartate transaminase (AST), was comprehensively analysed. In addition, the study assessed risk indicators including the prevalence ratio (PR) and odds ratio (OR) as well as the diagnostic accuracy of UHR and C-reactive protein (CRP) in detecting liver transaminases abnormalities, with analyses stratified by age and gender. *Results*: UHR was significantly elevated in subjects with increased ALT and AST activities, and this pattern was consistent across all age and gender categories. High UHR was significantly associated with elevated ALT (OR = 2.32, 95% CI: 2.12–2.53, *p* < 0.001) and AST (OR = 1.38, 95% CI: 1.25–1.52, *p* < 0.001), with stronger associations observed in males and for ALT activity. In addition, elevated UHR was more prevalent among individuals with increased liver transaminase activities. Receiver operating characteristic (ROC) analysis showed that UHR outperformed CRP in identifying elevated liver transaminases, with better discriminative ability for ALT than AST activity. *Conclusions*: These findings highlight a significant association between UHR and liver transaminase abnormalities in the general population, underscoring the potential utility of UHR as a simple and accessible indicator for liver function assessment in clinical settings.

## 1. Introduction

The liver is a vital organ involved in many physiological processes. It synthesizes numerous essential proteins implicated in the immunological response, blood clotting factors, erythropoiesis, digestive enzyme production, the storage and synthesis of glucose, and metabolic detoxification [1,2]. The liver enzymes alanine aminotransferase (ALT) and aspartate aminotransferase (AST) are key enzymes that are involved in gluconeogenesis by facilitating the transfer of amino groups from alanine or aspartic acid to ketoglutaric acid, resulting in the production of oxaloacetic acid and pyruvic acid [3,4]. Elevated levels of liver transaminases typically indicate hepatocellular injury and have been associated with several chronic conditions, including non-alcoholic fatty liver disease (NAFLD), cardiovascular disease, hypertension, type 2 diabetes, and chronic kidney disease [5,6,7,8,9]. Notably, multiple studies suggest that even transaminase levels within the upper range of normal may indicate an increased risk for metabolic disorders such as diabetes [10,11].

Purines are fundamental components in DNA and RNA molecules. During their metabolic breakdown, the enzyme xanthine oxidase catalyses the conversion of purine nucleotides into uric acid (UA), which represents the end product of purine catabolism [12]. The liver is the primary site of this biological process, where the enzyme xanthine oxidase is abundantly expressed [13]. Elevated serum UA levels have been linked to various chronic disorders such as coronary artery diseases, renal diseases, and gout [14,15,16].

High-density lipoprotein (HDL) particles, primarily produced in the liver and small intestine, are a key plasma lipoprotein and exhibit notable anti-inflammatory and antioxidant properties [17]. Normal levels of HDL-C, which is the cholesterol carried within HDL particles and traditionally referred to as “good cholesterol,” have been associated with a lower risk of metabolic disorders such as atherosclerosis and cardiovascular diseases [18]. Studies have linked low HDL-C with the severity and progression of non-alcoholic fatty liver (NAFLD) and chronic renal diseases [19,20]. Hence, enhancing HDL-C concentrations is considered a potential therapeutic strategy to reduce the risk of metabolic and chronic disorders [21,22,23,24].

The serum UA-to-HDL-C ratio (UHR) is one of the new-generation inflammatory markers and has been evaluated in the context of different health disorders such as ischemic heart disease, diabetic complications, thyroiditis, and hypertension [25,26,27,28]. Given the link between elevated uric acid, reduced HDL-C levels, and various metabolic dysfunctions, UHR may serve as a simple yet effective biomarker for detecting and monitoring liver transaminase abnormalities. It is known that healthcare facilities in certain areas of the world, particularly in large parts of the Middle East, lack reliable access to essential diagnostic resources due to various reasons. Therefore, generating simple, straightforward, and affordable markers to identify and monitor health disorders is very valuable. In this context, the current study presents the first extensive population-based investigation to assess the association between UHR and liver transaminase elevation.

## 2. Materials and Methods

### 2.1. Data Collection and Study Design

**Figure 1:**
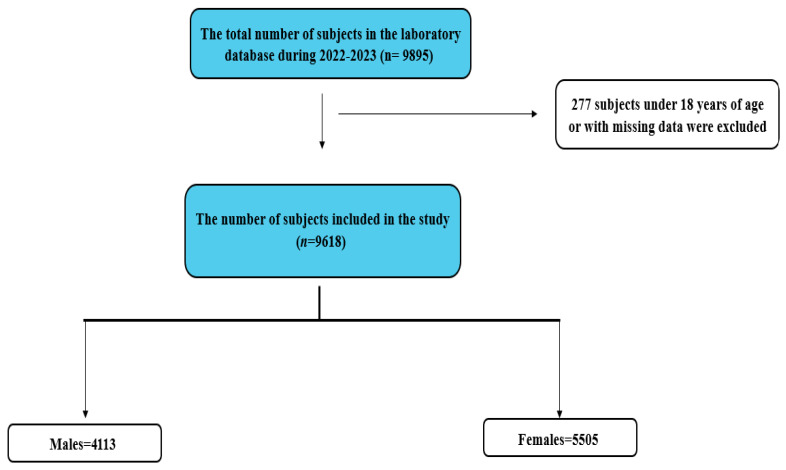
Schematic overview of the studied population.

Subsequent to Ethics Committee approval at King Saud University (Approval No. E-24-9466), the research team extracted the demographic and laboratory data of 9895 subjects from the ELTA laboratories database for the years 2022–2023, reflecting a broad cross-section of the adult general community, to conduct the present retrospective cross-sectional study. A total of 277 subjects who were under the age of 18 or with missing liver transaminases data were excluded (Figure 1). Due to the nature of the study and the structure of the database, additional exclusion criteria, such as comorbidities or medication history, were not applied. Importantly, based on the cultural and legal context of Saudi Arabia, where alcohol consumption is strictly prohibited and extremely rare, the study population is assumed to be free from alcohol-related liver effects.

The study population was classified based on gender and age. The Early Adults group contained participants aged between 18–29 years. The Middle-Aged Adults group included subjects aged between 30–44 years. The Older Adults group included participants older than 44 years. Overall, 42.76% of the studied population were males, whereas females represented 57.24% of the population, Table 1.

Elevated ALT and AST activities were defined according to the reference ranges reported by Borai et al. [29]. UHR was calculated manually as UA(mg/dL)÷HDL−C(mg/dL). A UHR of >11.08 was considered elevated, as determined by the optimal cutoff (i.e., highest sensitivity and specificity) using receiver operating characteristics (ROC) analysis. A CRP higher than 0.3 mg/dL was considered high [30].

### 2.2. Statistics

Since the studied data did not follow a normal distribution, statistical analysis was carried out using nonparametric approaches, with group comparisons conducted via the Mann–Whitney U test. The baseline data were summarised utilizing medians and interquartile ranges (IQR) for continuous data and percentages for categorical data. To evaluate diagnostic performance, ROC curve analysis was conducted, and the area under the curve (AUC) was calculated to determine the sensitivity and specificity. Risk assessment, including prevalence ratios, odds ratios, and 95% confidence intervals, was conducted using MedCalc Statistical Software version 20.218 (MedCalc Software Ltd., Ostend, Belgium). The statistical analyses were performed using GraphPad Prism v10.0.1 (GraphPad Software, Inc., San Diego, CA, USA), with a significant *p*-value set at <0.05.

## 3. Results

**Figure 2:**
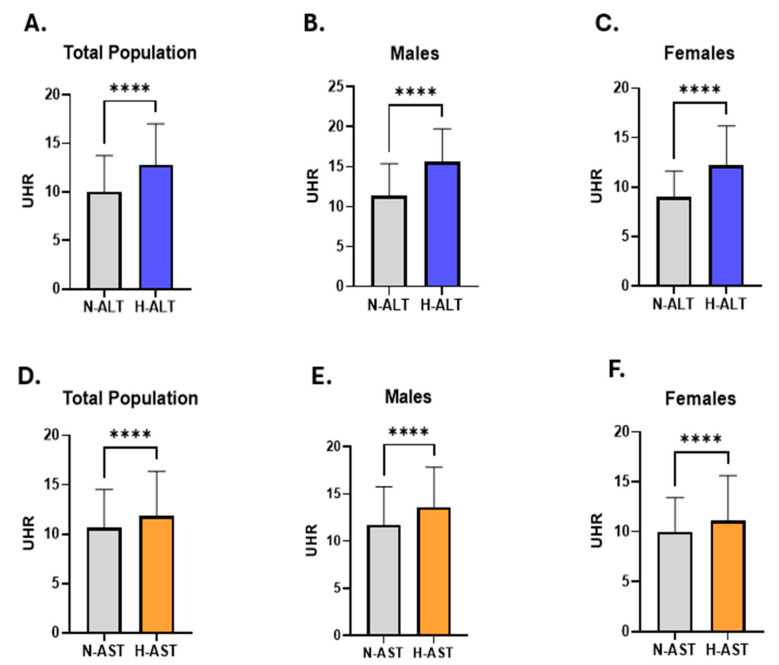
Comparison of UHR measurements between subjects with normal and elevated liver transaminases. The levels of UHR in N-ALT and H-ALT groups are illustrated for the total population (**A**), male subjects (**B**), and female participants (**C**). The difference in the pattern of UHR between N-AST and H-AST groups is shown for both genders (**D**), males (**E**), and females (**F**). Asterisks mark the significance level; **** for *p* < 0.0001.

To evaluate the relationship between the UHR and liver transaminase activities, we categorized the studied subjects according to their ALT and AST activities into four groups: Normal ALT group (N-ALT), high ALT group (H-ALT), normal AST group (N-AST), and high AST group (H-AST). As revealed in Figure 2, UHR was significantly increased in the H-ALT group (12.83: 9.19–17.01) compared to subjects with normal ALT activity (10: 7.460–13.75) (Figure 2A). This finding remained consistent when analysing males and females individually (Males; 15.62: 12.05–19.73 vs. 11.36: 8.14–15.37) (Females; 12.20: 8.81–16.19 vs. 9.02:6.96–11.63) (Figure 2B,C).

Similarly, when comparing UHR values between the N-AST and H-AST groups, we observed a rise in UHR in the H-AST (11.8: 8.37–16.36) in comparison to the N-AST group (10.63: 7.80–14.52) (Figure 2D). This pattern persisted upon separate analysis by gender (Males; 13.57: 9.41–17.85 vs. 11.70: 8.28–15.75) (Females; 11.09: 7.92–15.61 vs. 10: 7.50–13.40) (Figure 2E,F).

**Figure 3:**
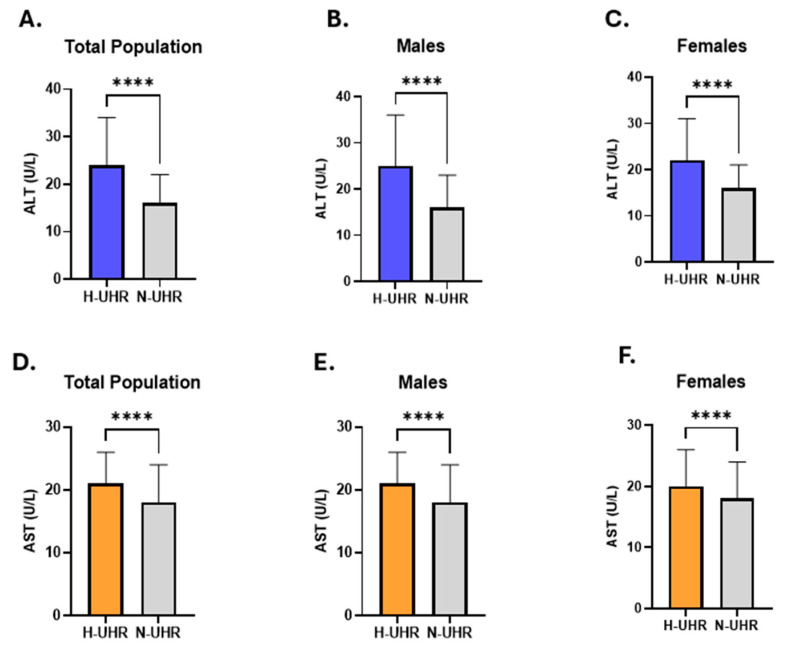
Evaluation of liver transaminase activities in relation to UHR across the entire population. A comparison of ALT levels in individuals with a high UHR compared to those with a normal UHR in the total cohort (**A**), among males (**B**), and females (**C**) is shown. The difference in AST patterns between participants with a high UHR compared to those with a normal UHR is revealed for all studied subjects (**D**), as well as in males (**E**), and females (**F**). Asterisks mark the significance level; **** for *p* < 0.0001.

Then, ALT activity in light of the UHR was evaluated in the study cohort. Our analysis showed that individuals with an elevated UHR had higher ALT activity (24: 17–34) than those with a normal UHR (16: 12–22) (Figure 3A). A comparable pattern was observed when each sex was examined independently (Males; 25: 18–36 vs. 16: 12–23) (Females; 22: 16–31 vs. 16: 12–21) (Figure 3B,C). In addition, we evaluated the AST activity in light of UHR and found that individuals with an elevated UHR had increased levels of AST activity (21: 17–26) compared to subjects with a normal UHR (18: 15–24) in the study population (Figure. 3D). Separate evaluation of the genders revealed a similar trend (Males; 21: 17–26 vs. 18: 15–24) (Females; 20: 17–26 vs. 18: 15–24) (Figure 3E,F).

**Figure 4:**
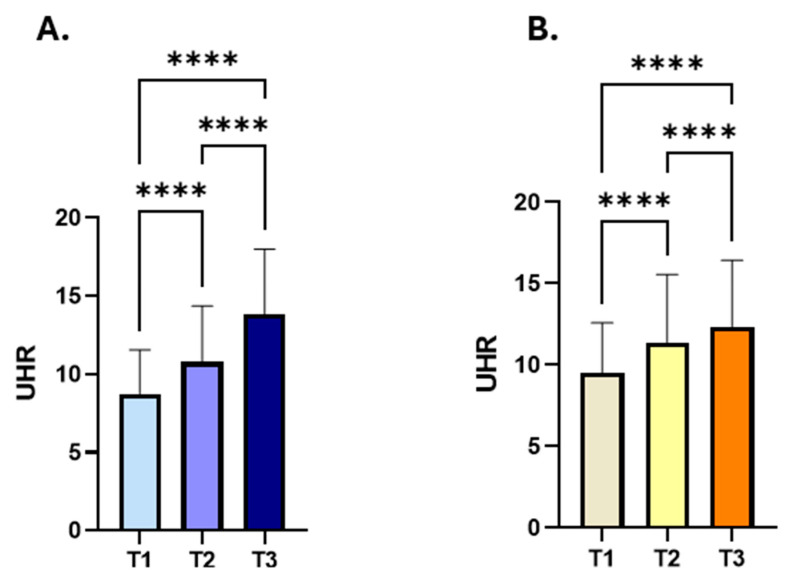
Comparison of UHR measures across liver transaminase tertiles in the studied subjects. Participants were categorized into three groups based on their liver transaminases activity (T1 = lowest, T2 = middle, T3 = highest). UHR levels progressively increased in accordance with increasing ALT (**A**) and AST (**B**) tertiles. Asterisks mark the significance level; **** for *p* < 0.0001.

To further assess the link between liver enzyme activity and the UHR, the subjects were stratified based on their liver transaminases into three tertiles, and UHR values were then analysed across these tertiles. Our findings showed a progressive increase in UHR levels in accordance with higher ALT (UHR; 8.75: 6.77–11.56 vs. 10.77; 8.178–14.37 vs. 13.85: 10–18) and AST (UHR 9.5: 7.14–12.57 vs. 11.37: 8.25–15.56 vs. 12.35: 8.71–16.43) activities (Figure 4A,B).

**Figure 5:**
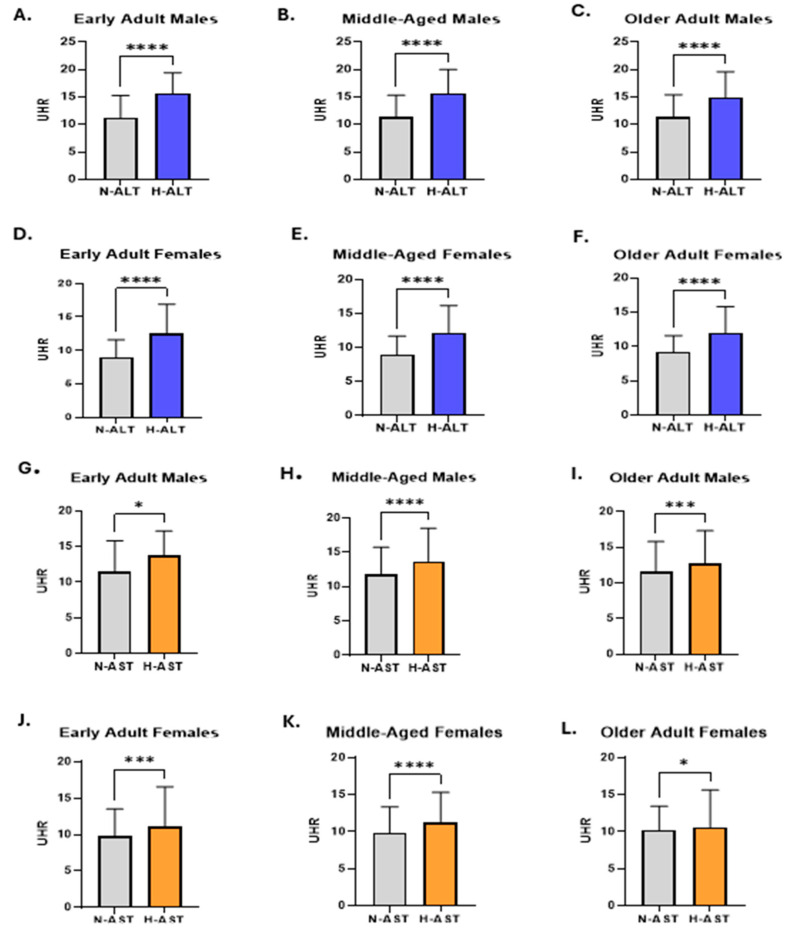
Combined age- and gender-based comparisons of UHR measures in relation to liver transaminase activities. The difference in UHR levels between males with N-ALT and H-ALT across early adults (**A**), middle-aged adults (**B**), and older adults (**C**) is shown. The difference in the UHR measures between N-ALT and H-ALT groups is shown for female early adults (**D**), middle-aged adults (**E**), and older adults (**F**). The levels of UHR in N-AST and H-AST groups are illustrated for male early adults (**G**), middle-aged adults (**H**), and older adults (**I**). The difference in UHR measures between N-AST and H-AST groups is revealed for female early adults (**J**), middle-aged adults (**K**), and older adults (**L**). Asterisks mark the significance levels: * for *p* < 0.05, *** for *p* < 0.001, and **** for *p* < 0.0001.

Subsequently, age- and gender-stratified comparisons were performed. The analysis showed UHR levels were significantly higher in H-ALT groups compared to those with normal ALT measures, consistently across all age categories in both sexes. Among males with high ALT activity, early adults (15.63: 12.31–19.42 vs. 11.29: 8.20–15.31), middle-aged adults (15.70: 12.30–20 vs. 11.33: 8.03–15.33), and older adults (14.91: 11.53–19.61 vs. 11.43: 8.30–15.43) exhibited significantly higher UHR measures compared to their counterparts with normal ALT activity (Figure 5A–C). A similar trend was observed in female participants with elevated ALT activity, where UHR levels were consistently higher across all age groups compared to those with normal ALT activity (Figure 5D–F). Additionally, the analysis revealed that UHR measures were significantly elevated in individuals with H-AST activity in comparison to those with normal N-AST, in both genders across all age groups (Figure 5G–L), confirming the consistent association between UHR levels and the increased activity of liver transaminase.

The distribution pattern of elevated UHR levels in the studied population was analysed. Overall, individuals with elevated AST activity exhibited a slightly higher proportion of high UHR levels (54.71%) compared to those with normal AST activity (45.29%), while elevated UHR levels were more frequently observed among participants with H-ALT activity (62.32%) than among those with ALT values within the normal range (41.57%); Table 2.

Among male participants, elevated UHR levels were more common in those with increased AST activity (64.31%) compared to those with normal AST levels (54.15%). Notably, the proportion was even higher in males with elevated ALT activity, reaching 80.82%, in contrast to 51.90% in those with normal ALT values. In female participants, the proportion of individuals with elevated UHR levels was higher among those with increased AST activity (50.04%) compared to those with normal AST levels (40.35%). Furthermore, our observation showed a greater prevalence of high UHR measures among female participants with elevated ALT activity (58.33%) compared to those with normal ALT levels (28.97%).

The relationship between elevated UHR and the risk of abnormal liver enzyme activities was evaluated in Table 3. The results revealed that a high level of UHR was associated with an increased risk of elevated ALT activity in the entire study population (PR = 1.49, 95% CI: 1.44–1.55, *p* < 0.001), in male participants (PR = 1.55, 95% CI: 1.47–1.63, *p* < 0.001), and females (PR = 2.01, 95% CI: 1.88–2.14, *p* < 0.001). Also, our analysis showed that elevated levels of UHR were linked with an increased risk of having abnormally high AST activity in both genders (PR = 1.17, 95% CI: 1.12–1.22, *p* < 0.001), in males (PR = 1.18, 95% CI: 1.11–1.26, *p* < 0.001), and in female subjects (PR = 1.24, 95% CI: 1.16–1.32, *p* < 0.001).

Furthermore, the analysis showed that the likelihood of male subjects with elevated UHR falling into the H-ALT group was 3.9 (95% CI: 3.13–4.87, *p* < 0.001), whereas the likelihood of females with increased UHR falling into the H-ALT group was 3.43 (95% CI: 3.06–3.83, *p* < 0.001). Male and female participants with elevated UHR levels had 1.52 (95% CI: 1.28–1.80, *p* < 0.001) and 1.48 (95% CI: 1.31–1.67, *p* < 0.001)-times the chance of having elevated AST activities, respectively.

**Figure 6:**
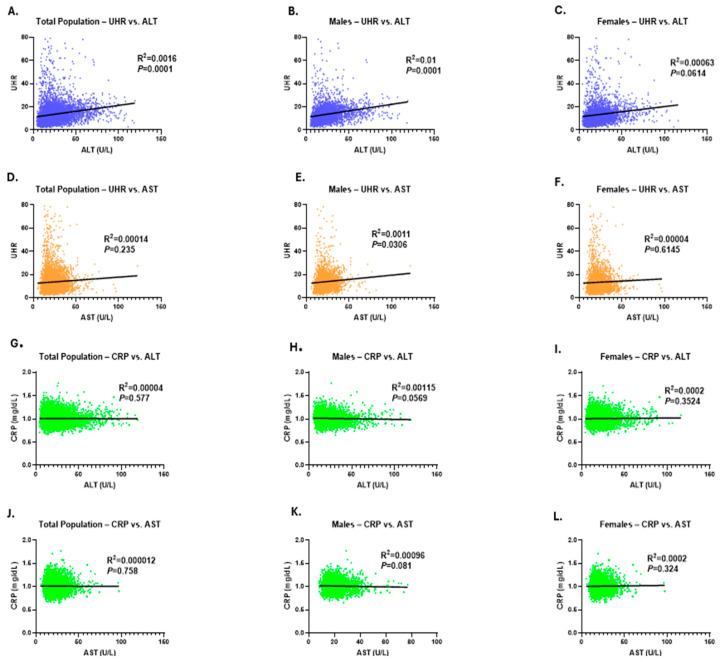
Simple linear regression of the association between UHR measures and transaminase activities. The correlation between UHR levels and ALT activity is shown for the total study population (**A**), in male subjects (**B**), and females (**C**). The correlation between UHR measures and AST levels in both genders (**D**), in males (**E**), and female subjects (**F**). Simple linear regression of association between CRP and ALT in the total study population (**G**), in male subjects (**H**), and females (**I**). The correlation between CRP measures and AST levels in both genders (**J**), in male participants (**K**), and female subjects (**L**).

As demonstrated in Figure 6, the correlation between UHR measures and liver transaminase activity was evaluated. The results showed a marginal positive correlation between ALT levels and UHR in the studied cohort, with this association being more apparent among male subjects (Figure 6A,B). In addition, UHR showed a weak correlation with AST activities in male participants (Figure 6E). In contrast, no significant association was detected between CRP concentrations with ALT or AST activities across both genders (Figure 6G–L). These findings imply that although UHR does not exhibit a direct correlation with ALT and AST activities, it may influence them indirectly through other mediating factors involved in the underlying biological processes.

A regression analysis was performed to further explore the relationship between UHR and potential predictors, including age, gender, and liver transaminase activity, Table 4. The analysis showed a significant effect of ALT activity on the UHR measure (β = 0.1304; 95% CI: 0.0654–0.1954; *p* < 0.001). Age and gender were not significant contributors to the changes in UHR levels. These findings further confirm the association between UHR and liver transaminase activity, particularly with ALT.

**Figure 7:**
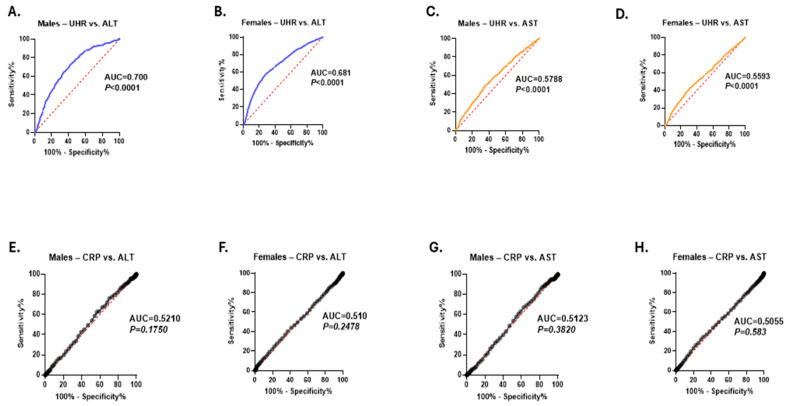
The diagnostic performance of UHR and CRP in discriminating elevated liver transaminase activities. ROC curve of UHR to discriminate elevated ALT activity in males (**A**) and females (**B**). ROC curve of UHR to discriminate elevated AST levels in males (**C**) and females (**D**). ROC curve of CRP to discriminate high ALT in males (**E**) and females (**F**). ROC curve of CRP to discriminate high AST in males (**G**) and females (**H**).

Additionally, to provide a more comprehensive and comparative assessment of the clinical utility of UHR and CRP in distinguishing individuals with elevated liver transaminase levels from those with normal enzyme activities, the ROC curve test was conducted separately for males and females. The analysis showed a significantly stronger discriminative performance of UHR for elevated ALT activities compared to CRP in both males (UHR: AUC = 0.700, *p* < 0.001 vs. CRP: AUC = 0.521, *p* = 0.175) and females (UHR: AUC = 0.681, *p* = 0.001 vs. CRP: AUC = 0.51, *p* = 0.247) (Figure 7A,B,D,E). In addition, UHR also showed a modest advantage over CRP in distinguishing individuals with high AST activity in both genders (Figure 7C,D,G,H).

## 4. Discussion

UHR is one of the new generation inflammatory markers that are implicated in several metabolic and health conditions [25,27,28]. However, studies investigating the link between UHR with liver transaminase abnormalities remain limited. This study reports novel findings demonstrating a significant association between UHR measures and elevated ALT and AST activities among a large cohort of Saudi general adults. Furthermore, the results of this investigation showed a superior diagnostic utility of UHR in identifying liver transaminase abnormalities in comparison to the classical inflammatory marker CRP. To the best of our knowledge, this is the first population-based study to assess this association within a general community setting.

Although males and females have different normal ranges of liver transaminases [31], the current investigation reported a significant increase in UHR levels in subjects with elevated ALT and AST activities in both sexes. This result suggests that UHR could be a good diagnostic marker to monitor ALT and AST activities despite the gender-based variations in hepatic transaminase. Additionally, stratified by liver transaminase activity, the results of this study showed that individuals with elevated UHR levels exhibited a significant increase in ALT and AST measures compared to those with normal UHR levels, across all ages and genders.

The findings of this study are consistent with previous reports linking UHR to liver dysfunction. A study by Li et al., showed a positive association between elevated ALT activity and UHR in a cohort of adolescents and children with short stature, which supports the broader relationship observed in the current investigation across various age groups [32]. Another study involving 117 participants linked the increase in UHR levels with the elevation of ALT and other metabolic abnormalities, suggesting a potential utility of UHR as a marker for non-alcoholic hepatic steatosis [33]. Similarly, a study involving sixty NAFLD patients demonstrated a significant increase in UHR levels compared to healthy controls, with a positive correlation observed between liver transaminase activities and UHR [33]. Ou et al. reported an independent association of UHR with all-cause mortality in patients with metabolic dysfunction-associated steatotic liver diseases [34]. Moreover, a U.S.-based study reported a link between high UHR levels and NAFLD, suggesting the potential utility of UHR as a practical marker for identifying and monitoring the disease progression [35].

The UHR is a combination of UA and HDL-C. Therefore, elevated UHR can be attributed to a reduction in HDL-C levels, an increase in UA levels, or both. Multiple studies suggested a positive association between UA and hepatic disorders in different populations [36,37,38]. In a prospective cohort study involving 2832 subjects from the Chinese population, Wei et al. found a positive correlation between elevated UA levels and the incidence of NAFLD. In addition, that study showed an increase in the UA associated with a significant elevation in ALT activity in the studied population and suggested that UA can be used as a marker to predict NAFLD [39].

Gout is a condition characterized by increased accumulation of UA in the body. Multiple investigations have demonstrated an association between gout with metabolic and liver diseases [38,40]. A prospective study involving 113 subjects suggested that hyperuricemia is an independent risk factor for NAFLD and liver fibrosis in the Indonesian population [41]. Elevated levels of UA can potentially cause liver damage through different mechanisms. UA can induce oxidative stress and mitochondrial dysfunction in hepatocytes either directly or by disrupting the metabolic processes of glucose [42]. In addition, increased UA has the potential to enhance lipid synthesis through its ability to stimulate endoplasmic reticulum (ER) stress and activate Sterol regulatory element-binding protein 1 (SREBP-1) and other transcriptional factors, which could eventually cause chronic inflammation and liver damage [43], leading to the subsequent release of ALT and AST into the bloodstream.

It has been suggested that dyslipidemia is associated with increasing risks of various chronic illnesses such as atherosclerosis and cardiovascular disease [44,45]. A prospective observational study included 457 subjects by Cordero et al. showed an independent association between low HDL-C and the risk of acute coronary disease [46]. Contradictory findings have been reported by a number of studies regarding the correlation between low HDL-C and the liver’s transaminase activity and function. An investigation led by Jiang et al. reported that high HDL-C levels were associated with elevated liver transaminase activities [47]. Nevertheless, it is widely accepted that low HDL-C is associated with increased ALT and AST activities and liver diseases. For instance, a study by Trojak et al. linked low HDL-C with the development of NAFLD in patients with type 2 diabetes [48]. Similarly, another report demonstrated that reduced levels of HDL-C and its primary component, apolipoprotein A-I, were associated with greater disease severity, complications, and poorer survival outcomes in patients with liver failure [49]. Chronic inflammation is known to produce a variety of prooxidants that cause the oxidation of low-density lipoprotein (LDL) [50]. However, it has been suggested that HDL particles possess significant antioxidative and anti-inflammatory properties, allowing them to protect LDL from oxidative stress. Studies have shown that HDL transports enzymes, such as paraoxonase and platelet-activating factor acetylhydrolase, that have a role in inhibiting LDL oxidation [17,51]. It has been reported that the abnormalities of the lipid profile are associated with the induction of inflammation and oxidative stress in hepatocytes, leading to increased hepatic enzyme activities, including ALT and AST, and their subsequent release outside [43,52].

One of the notable findings in this investigation is that the association between UHR and liver enzyme activity was more apparent with ALT than with AST. These results could be attributed to a longer half-life of ALT than AST in circulation. In addition, while ALT is mainly found in hepatocytes, AST is diffused into other organs such as the skeletal muscles, heart, kidneys, and RBCs, making ALT more specific for liver injury than AST [53,54]. Furthermore, the findings of our investigation showed a stronger association of UHR with ALT in males compared to females. This observation could be attributed to the hormonal variations between genders, especially the influence of testosterone, as males typically have higher uric acid levels and lower HDL-C concentrations, leading to enhanced sensitivity of ALT activity to changes in UHR measures [55,56].

CRP, a general marker of acute inflammation, is known to be associated with inflammatory conditions. It has been shown that CRP is significantly increased during the acute phase of viral hepatitis diseases [57,58,59]. The findings of this study revealed that UHR exhibited a relatively stronger correlation with both ALT and AST activity compared to CRP. Moreover, UHR significantly outperformed CRP in identifying individuals with elevated liver transaminases, particularly ALT. Given its classification as an acute-phase protein, it is plausible to assume that CRP may demonstrate a reduced sensitivity to discriminate chronic conditions, such as the gradually increased activity of hepatic transaminases.

To our knowledge, this is the first and most comprehensive population-based study to examine the relationship between UHR and liver transaminase abnormalities in the general population of Saudi Arabia or any other Middle Eastern nation. This study has numerous advantages, such as using a substantial sample size to ensure the general population is adequately represented, and automated data acquisition, which decreases analytical variability. Nevertheless, due to the cross-sectional nature of this investigation, it was not possible to determine the causality between the UHR and liver transaminase abnormalities. Additionally, there was a lack of other potential confounding variables, such lifestyle and medication use, as the studied population was derived from a general community-based dataset.

## 5. Conclusions

Altogether, this report showed a positive association between elevated liver transaminases and the UHR in the general Saudi population, with a stronger association observed for ALT compared to AST. Considering the global impact of liver disease and the restricted availability of advanced diagnostic tools in many areas, especially in Middle Eastern countries, the UHR may offer a simple and accessible biomarker to aid in the early identification and ongoing monitoring of liver dysfunction within public health settings.

These findings encourage further longitudinal studies to evaluate the potential ability of the UHR as a diagnostic and prognostic marker for liver enzyme abnormalities and diseases in different populations, with more information on potential confounding factors.

## Figures and Tables

**Table 1:** Distribution of enrolled participants.

Gender	Number of Subjects (%)	Median UHR (IQR)
Total population	9618	10.83 (7.920–14.91)
Male subjects	42.76%	11.96 (8.50–16.14)
Early Adults	5.88%	12.02 (8.495–15.99)
Middle-Aged Adults	22.65%	12.04 (8.360–16.17)
Older Adults	14.22%	10.83 (7.920–14.91)
Female subjects	57.24%	10.20 (7.620–13.85)
Early Adults	10.55%	10.20 (7.780–13.90)
Middle-Aged Adults	33.24%	10.19 (7.50–13.83)
Older Adults	13.44%	10.23 (7.73–13.86)

Abbreviations: UHR, uric acid to HDL-cholesterol ratio; IQR, interquartile range.

**Table 2:** Prevalence of elevated liver transaminase in relative to the UHR measures.

Parameter	N-ALT Group	H-ALT Group	N-AST Group	H-AST Group
Both gender				
H-UHR	41.57%	62.32%	46.63%	54.71%
N-UHR	58.43%	37.68%	53.37%	45.29%
Males				
H-UHR	51.90%	80.82%	54.15%	64.31%
N-UHR	48.10%	19.18%	45.85%	35.69%
Females				
H-UHR	28.97%	58.33%	40.35%	50.04%
N-UHR	71.03%	41.67%	59.65%	49.96%

Abbreviations: ALT, alanine transaminase; AST, aspartate transaminase; UHR, uric acid to HDL-cholesterol ratio; H-UHR, high UHR; N-UHR, normal UHR; H-ALT, high ALT; N-ALT, normal ALT; H-AST, high AST; N-AST, normal AST.

**Table 3:** Risk assessment of increased UHR and elevated liver transaminase activities.

		Score	95% CI	Z Statistic	*p*-Value
	PR				
	Both genders	1.49	1.44–1.55	20.009	<0.001
	Males	1.55	1.47–1.63	16.915	<0.001
	Females	2.01	1.88–2.14	20.937	<0.001
	OR				
ALT	Both genders	2.32	2.12–2.53	18.913	<0.001
	Males	3.9	3.13–4.87	12.096	<0.001
	Females	3.43	3.06–3.83	21.613	<0.001
	PR				
	Both genders	1.17	1.12–1.22	6.807	<0.001
	Males	1.18	1.11–1.26	5.268	<0.001
	Females	1.24	1.16–1.32	6.555	<0.001
AST	OR				
	Both genders	1.38	1.25–1.52	6.505	<0.001
	Males	1.52	1.28–1.80	4.846	<0.001
	Females	1.48	1.31–1.67	6.294	<0.001

Abbreviations: PR, prevalence ratio; OR, odds ratio; CI, confidence interval; ALT, alanine transaminase; AST, aspartate transaminase; UHR, uric acid to HDL-cholesterol ratio.

**Table 4:** Regression analysis of UHR and selected predictors.

Variable	Estimate (β)	95% CI	*p*-Value
Age	−0.0338	−0.1103 to 0.0428	0.3868
Gender	0.1875	−1.321 to 1.696	0.8075
ALT	0.1304	0.0654 to 0.1954	<0.001
AST	−0.0823	−0.1953 to 0.0308	0.1537

## Data Availability

The data presented in this study are available on request from the corresponding author.
